# Ontogeny of melanophore photosensitivity in rainbow trout (*Oncorhynchus mykiss*)

**DOI:** 10.1242/bio.201410058

**Published:** 2014-10-10

**Authors:** Shyh-Chi Chen, R. Meldrum Robertson, Craig W. Hawryshyn

**Affiliations:** 1 Department of Biology, Queen's University, Kingston, ON K7L 3N6, Canada; 2 Centre for Neuroscience Studies, Queen's University, ON K7L 3N6, Canada, Canada

**Keywords:** chromatophore, photoreceptor, opsin, photoresponse, pigment cell, rainbow trout, visual pigment

## Abstract

Migratory species experience morphological and physiological changes during transitions between different life stages. In particular, modification of sensory systems is critical for animals to adapt to new environments. For example, to prepare for entry into seawater, salmonids undergo smoltification with dramatic changes in ultraviolet photoreceptors and polarized vision, which are important for orientation and foraging behaviours. Extraretinal organs are also involved in photoreception; however, the ontogenetic development of extraretinal photoreceptors is not well known, especially in migratory species. Here, we investigated whether rainbow trout dermal photoreceptors, melanophores, undergo change in spectral sensitivity during smoltification and which candidate molecules may account for this ontogenetic alteration. Our results showed that, contrary to parr melanophores which are insensitive to light, smolt melanophores displayed chromatic photoresponses with the emergence of cryptochrome and melanopsin expression. We suggest that these modifications may benefit the active foraging behaviour of smolts and enable adaptation to variable environments.

## INTRODUCTION

Non-mammalian animals have multiple types of photoreceptors to detect changes in ambient light conditions allowing them to accomplish a variety of photosensory tasks, including both image- and non-image-forming processes (Peirson et al., 2009). Ontogenetic changes in photosensory systems enable organisms to adapt to their environments. This is particularly true and critical for migratory species whose environments can vary considerably at different life stages. Salmonids have been used as model organisms to study ontogenetic changes of the visual system and polarization sensitivity of vision (Hawryshyn, 2010). In *Oncorhynchus mykiss*, the appearance of UVS (ultraviolet-sensitive) cone photoreceptors leads to polarized UV visual sensitivity, which is important for plankton-foraging and orientation behaviours (Hawryshyn, 2010). On the other hand, extraretinal photoreceptors play an important role in non-visual functions. For example, the vertebrate pineal gland, the most investigated extraretinal photosensitive organ, is important for setting biological rhythms and modulating neurotransmission during development (Shand and Foster, 1999). Although the expression of visual pigments in extraretinal photoreceptors and the associated photoresponses have been reported (Shand and Foster, 1999), little is known about ontogenetic changes of such photoreceptors in peripheral tissues of migratory species.

Chromatophores are specialized dermal photosensitive pigment cells and are responsible for teleost pigmentation and color patterns. Based on their internal structure and pigments, chromatophores can be categorized into two major cell types with characteristics of light absorption (melanophores, erythrophores, xanthophores, and cyanophores) or light reflection (leucophores and iridophores) (Fujii, 1993). Teleosts can tune their coloration using two processes: long-term morphological color change through the adjustment of cell size or number, and short-term physiological color change via the movement of internal elements (Fujii, 1993). In addition to hormones, light can trigger rapid color change through the movement of pigment granules or crystal plates. Although ontogenetic changes of the visual system have been well-studied in different salmonids, how extraretinal photosensory systems of migratory species develop at different life stages remains poorly understood. In the dermal photosensory system of rainbow trout *Oncorhynchus mykiss*, we hypothesize that melanophore photosensitivity differs before and after smoltification, which is a period of dramatic change taking place in the visual system. To test our hypothesis, we investigated photoresponses of melanophores in parrs and smolts. We also examined the expression of candidate photopigments which could be involved in the photosensitivity of melanophores. Melanophore photosensitivity and photopigment expression in integumentary tissues differ in parrs and smolts, suggesting that the dermal photosensory system is important for adaptation to changes in the photic environment associated with migration. For the first time, our work demonstrates ontogenetic change of chromatophore photoresponses in migratory species, particularly with the possible involvement of two non-visual pigments in the extraretinal photosensory system. Furthermore, the rainbow trout melanophore is the second type of chromatophore with a chromatically-dependent antagonistic photosensitive mechanism besides the erythrophores of tilapia (*Oreochromis niloticus*) (Chen et al., 2013).

## RESULTS AND DISCUSSION

**Fig. 1.**
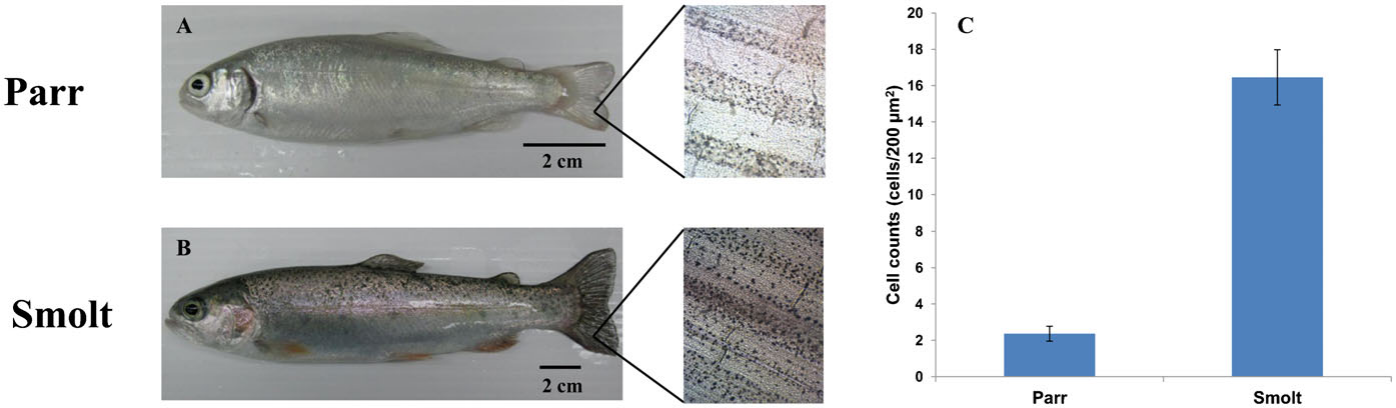
Pigmentation and melanophore counts of rainbow trout parr and smolt caudal fins. Pigmentation of (A) parr and (B) smolt. In close-ups of the caudal fins on the right of the images, note that in smolts the number of melanophores increased and more melanophores were present in the area between fin rays. (C) The number of melanophores in the area between fin rays increased in smolt (n = 11 for each group; also see supplementary material Table S1). Scale bars: 2 cm.

Many salmonids undergo morphological and physiological changes during ontogeny. In particular, smoltification accomplishes the transformation from the parr that is essential for an anadromous species prior to its seaward migration. Modulation of sensory systems is critical for adaptation to distinct habitats at different life stages. Chromatophores are the primary agents contributing to body coloration and mediating body coloration changes in response to light. Therefore, any morphological or physiological change of chromatophores, such as cell numbers or the ability to translocate pigment granules, will lead to the modifications of body patterns and pigmentations. In the present study, we aimed at understanding how the dermal photosensory system of migratory species develops at different life stages. First, we investigated ontogenetic changes of melanophore distributions in parrs and smolts of rainbow trout (*Oncorhynchus mykiss*). We found that the patterns of melanophore distribution in caudal fin tissues of parrs and smolts were different. In parrs, most melanophores were restricted to the regions near fin rays (Fig. 1A). During smoltification, melanophores increased in number and extended their distribution to the region between fin rays. Thus, smolts had darker caudal fins with more melanophores, notably in the area between fin rays (Fig. 1B,C). Eventually, the development of melanophores leads to morphological color change of *O. mykiss*.

**Fig. 2.**
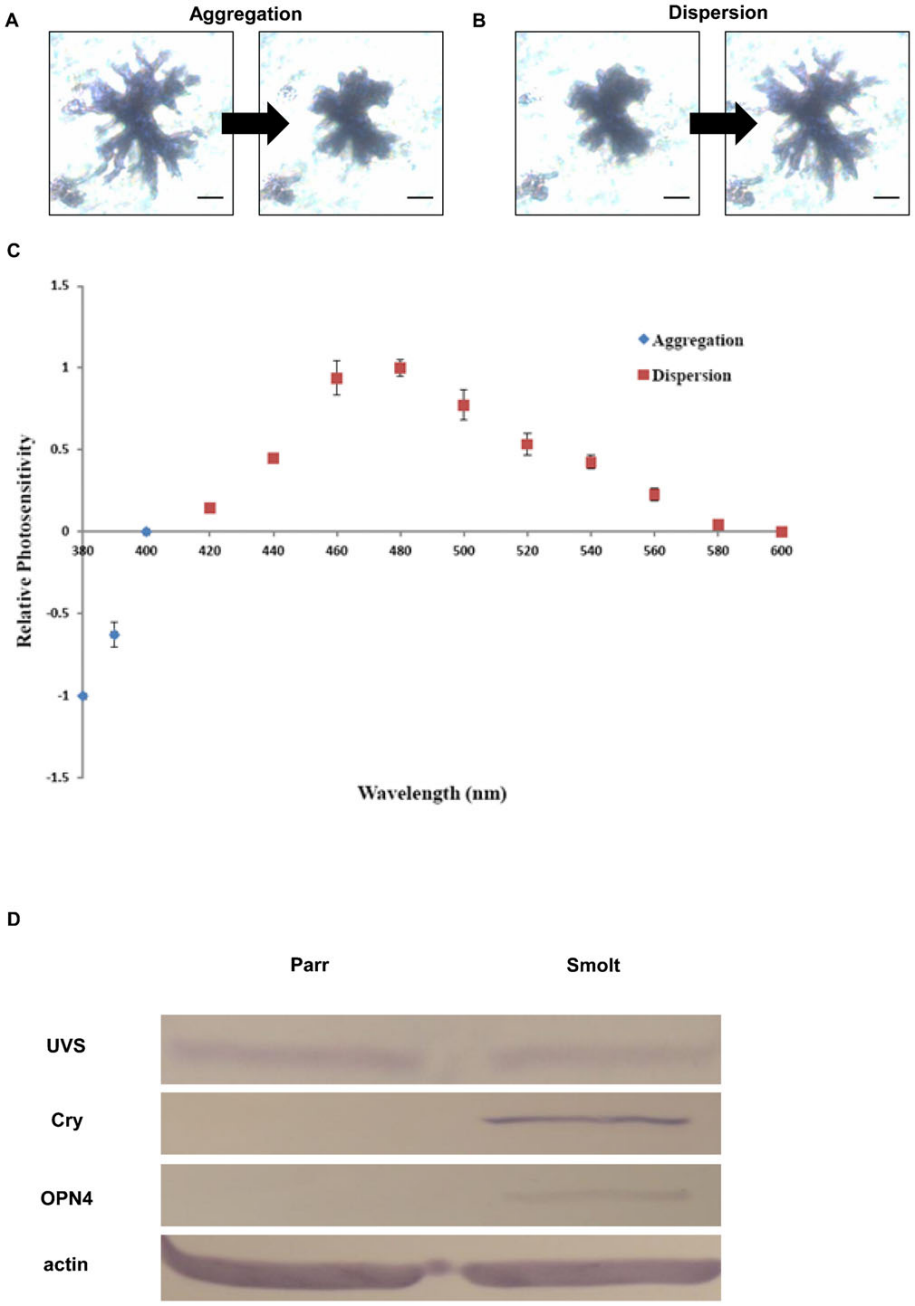
Spectral sensitivity of smolt melanophores and candidate photopigments involved in melanophore photosensitive processes. (A,B) Incident light triggered a smolt melanophore to aggregate or disperse inner pigment granules (melanosomes): (A) aggregation at 380 nm; (B) dispersion at 480 nm. (C) Melanophores responded to light in a wavelength-dependent manner. Aggregations occurred with UV and short wavelength illumination and dispersions with middle and long wavelength illumination. (D) Western blot analysis of photopigment proteins of the integumentary tissues of parrs and smolts. Proteins extracted from caudal fins of parrs and smolts were subjected to western blot analysis. Blots were probed with antibodies against UVS, Cry, OPN4, and actin which served as a loading standard. After incubation with NBT/BCIP, UVS, Cry, and OPN4, and actin were detected at their expected sizes (in kDa): UVS = ∼40; Cry = ∼66; OPN4 = ∼63; actin = ∼42. Scale bars: 50 µm.

Subsequently, we examined if melanophore photoresponses undergo ontogenetic change during smoltification. Under illumination, parr melanophores did not show obvious photoresponse regardless of wavelengths (n = 16; supplementary material Fig. S1); Conversely, light triggered smolt melanophores to aggregate or disperse their pigment granules, melanosomes (Fig. 2A,B). Smolt melanophores showed photoresponses in a wavelength-dependent manner. Aggregations occurred in the UV (380–400 nm) region and dispersions at 420–600 nm (n = 8; Fig. 2C). Furthermore, the spectral sensitivity curve showed two response peaks at 380 and 480 nm (Fig. 2C), suggesting that two light-sensitive molecules could be involved in the photoresponses: one photopigment being responsible for aggregation in the UV and short wavelengths and the other for dispersion in the middle and long wavelengths. This biphasic photosensitivity of rainbow trout melanophores is similar to the spectral characteristics of many extraretinal photoreceptors showing sensitivity to short- and middle-wavelength regions (Shand and Foster, 1999).

Chromatophore photoresponses are associated with photopigment expression (Ban et al., 2005). In the spectral sensitivity curve of rainbow trout melanophores, we found distinct sensitivity peaks at 380 and 480 nm. In rainbow trout, opsins expressed in rods (RH1) and four classes of cones (UVS, SWS, MWS, and LWS) and their peak sensitivities (λ_max_) have been identified using different approaches: rods (RH1: 504 nm); UVS (SWS1: 373 nm), SWS (SWS2: 423 nm), MWS (RH2b: 500 nm; RH2a: 542 nm), and LWS (LWS: 585 nm) cones (Anderson et al., 2010; Hawryshyn et al., 2001). In addition to these retinal visual pigments, the possible involvement of novel light-sensitive molecules for non-visual functions in a variety of extraretinal photoreceptors cannot be excluded. For example, cryptochromes (Cry) are sensitive to short wavelengths, and melanopsin (OPN4) and vertebrate ancient (VA) opsins possess λ_max_ close to 480 nm, with a spectrum ranging from 460 to 500 nm (Peirson et al., 2009).

The sensitivity peak we found in the UV region makes UVS and cryptochrome the candidate photopigments responsible for aggregations. On the other hand, because a variable vitamin A1/A2 chromophore ratio can lead to spectral tuning of the peak absorbance of visual pigments (Hawryshyn et al., 2001), opsins like RH1, RH2b, OPN4, and VA, with peak absorption near 480 nm may be the opsin responsible for dispersions. In order to determine which photopigments could be involved in melanophore photoresponses, antibodies against three retinal opsins and one cryptochrome (UV-/short wavelength-sensitive UVS opsin and cryptochrome for aggregation, and middle wavelength-sensitive rhodopsin [RH1] and melanopsin for dispersion) were used in western blot analyses. We did not detect RH1expression in the integumentary tissues of parrs and smolts (data not shown). UVS was expressed at both stages though the expression level showed no obvious change associated with smoltification (Fig. 2D). Instead, the immunosignals of Cry and OPN4 were detected only at the smolt stage (Fig. 2D). Together, these results indicate that cryptochrome and melanopsin could be involved in the photoresponses of smolt melanophores, suggesting that cryptochrome and melanopsin are responsible for light-induced aggregation and dispersion, respectively. However, since the cryptochrome and melanopsin sequences of rainbow trout are not available, we are unable to validate their subtypes. Future research on mRNA expression at the single-cell level and functional analysis of photopigments will be helpful to understand the photosensory mechanism of melanophores.

Salmonids show variable foraging and prey-search behavior on both temporal and spatial scales. Their growth and mortality risks are greatly affected by the biotic and abiotic factors that are highly associated with the habitats at different life stages. Parrs tend to hold positions near the streambed and behave as “sit-and-wait” predators. They frequently feed on benthic invertebrates or by catching drifting prey locally. Instead, smolts show increased feeding activity and are able to capture a variety of surface prey. Thus, salmonids have to progressively adjust or tune their sensory systems during ontogeny. The distribution of chromatophores leads to the formation of pigment spots and bars; hence, the color change of chromatophores in response to light will certainly influence fish appearance. Although the change of coloration during the course of smoltification has been reported (Hoar, 1988), the ontogeny of chromatophore photoresponses in rainbow trout remains unknown so far. In the present study, rainbow trout melanophores showed photoresponses only in smolts but not parrs. For smolts, light-induced color change benefits their adaptation to different environments. In rainbow trout, the ontogenic change of body coloration and the appearance of retinal UVS cone are mediated by thyroid hormone (TH) (Hoar, 1988). It is possible that the development of intrinsic photosensitivity of melanophores is also a TH-regulated process. In order to fully evaluate the role of melanophore photoresponses in non-image-forming tasks during ontogeny, the characterization of melanophore photosensitivity at later life stages and the association with environments and behaviours must be determined. Nevertheless, our findings provide the first demonstration of developmental change of chromatophore photosensitivity of migratory species as well as developmental changes in the expression of two non-visual pigments, cryptochrome and melanopsin, which could participate in the photoresponses of extraretinal photoreceptors.

## MATERIALS AND METHODS

### Animals

Rainbow trout (*Oncorhynchus mykiss*) at two developmental stages were obtained from the Rainbow Springs Trout Hatchery (Thamesford, Ontario, Canada). Parrs were 13.5±3.8 g in weight and 10.2±1.7 cm in length; smolts were 88.45±3.4 g in weight and 21.1±0.3 cm in length. They were maintained in flow-through freshwater (15°C) holding tanks under a 12 h:12 h L:D light cycle. Lighting was provided by full spectrum fluorescent lamps (Full Spectrum Solutions, Inc., Jackson, MI, USA). Fish were anaesthetized by immersion in MS-222 (Syndel Laboratories Ltd., Qualicum Beach, BC, Canada) and split-fin tissues were taken from tails for the measurements of melanophore photoresponses. All procedures complied with the Canadian Council for Animal Care regulations.

### Measurements of cell numbers and photoresponses of melanophores

Split-fin tissues containing melanophores were perfused with PBS (15°C; NaCl 125.3 mM, KCl 2.7 mM, CaCl_2_ 1.8 mM, MgCl_2_ 1.8 mM, D-glucose 5.6 mM, Tris-HCl buffer 5.0 mM [pH 7.2]; Sigma–Aldrich [Oakville, Ontario, Canada]) and were stimulated with light generated by a 150 W xenon lamp system and a monochrometer (Photon Technology International, London, ON, Canada). Images were taken using a Qimaging Microimager II CCD camera with QCapture Suite V2.46 software (Qimaging, Burnaby, BC, Canada). Cell counting was conducted in regions (200 µm^2^) between the 3^rd^ and 4^th^ fin rays. To measure melanophore photoresponses, Matlab software (Mathworks, Natick, MA, USA) was used for pixel counts of the pigment-covered area of a cell in a series of images. First, a cell was selected with a rectangular software tool, fitting it as close to the cell dendrites as possible. The selected image was converted to a binary image using a threshold level which defined pixel numbers with the highest correlation to the results obtained using Adobe Photoshop CS (Adobe Systems, San Jose, California) with manual selection (supplementary material Fig. S2). Pixel intensity values greater than the threshold were determined a value of 1. The sum of values in each image was used to represent the pigment-covered area within a melanophore.

Smolt melanophores displayed biphasic photoresponses, i.e. aggregations and dispersions, when presented with UV/short and middle/long wavelengths, respectively. To choose an appropriate intensity of stimulus, response versus intensity (RI) curves were generated for aggregation (at 380 nm) and dispersion (at 500 nm). For each measurement cycle, tissues were illuminated at 380 nm (13.88 log photons cm^−2^ s^−1^) or 500 nm (13.92 log photons cm^−2^ s^−1^) for 1.5 minutes, followed by 5-min darkness to reach full aggregation or dispersion. Subsequently, stimulus intensity was increased in a step-wise fashion with 1.5 minutes at each level controlled by a neutral density (ND) wedge (for aggregation: 12.26 [1.6ND], 12.62 [1.2 ND], 13.09 [0.8ND], 13.48 [0.4ND] and 13.88 [0ND] log photons cm^−2^ s^−1^; for dispersion: 12.87 [1ND], 13.09 [0.8ND], 13.3 [0.6ND], 13.52 [0.4ND], 13.72 [0.2ND] and 13.92 [0ND] log photons cm^−2^ s^−1^). The intensities (I_s_) required for half-maximal photoresponses were used for the following measurements of photoresponses (aggregations: 12.90 log photons cm^−2^ s^−1^; dispersions: 13.32 log photons cm^−2^ s^−1^).

To determine if cells were sensitive to light, melanophore photoresponses were measured as follows. First, full aggregation (A_full aggregation_) or full dispersion (A_full dispersion_) was achieved under illumination at 380 (13.88 log photons cm^−2^ s^−1^) or 500 nm (13.92 log photons cm^−2^ s^−1^) for 1.5 minutes, followed by 5-min darkness. Then, cells were presented with light stimulus at wavelengths (λ = 380, 420, 460, 500, 540, and 580 nm) at I_s_ for 1.5 minutes. The ratio (R) of pigment covered area change was calculated as:
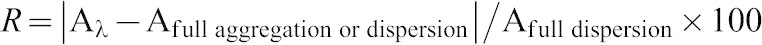
(1)where A_λ_ denotes the pixel counts of pigmented area at a particular wavelength (λ). We found that parr melanophores were insensitive to light, while smolt cells showed photoresponses at each test wavelengths (supplementary material Fig. S1). We further investigated the spectral sensitivity of smolt melanophores using the aforementioned method with smaller measuring interval from 380 to 600 nm (λ = 380, 390, 400, 420, 440, 460, 480, 500, 520, 540, 560, 580, and 600 nm). Melanophore photosensitivity (A_s_) was calculated as:
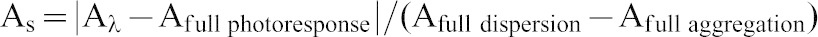
(2)A_full photoresponse_ is A_full dispersion_ when λ = 380, 390, 400 nm, and A_full aggregation_ when λ = 420–600 nm. These data were normalized with maximum aggregation and dispersion at −1 and +1, respectively, to minimize the variation between cells, and a photosensitivity curve of mean normalized sensitivity against wavelength was plotted.

### Western blot

Western blotting was performed using rat polyclonal anti-rainbow trout UVS (SWS1) opsin (Allison et al., 2006), rabbit polyclonal anti-zebrafish rod opsins (86% identity with the homolog of RH1 of *O. mykiss*; generous gifts from Dr. David Hyde, University of Notre Dame), mouse monoclonal anti-human cryptochrome 1 (Cry 1; Abcam Inc., Toronto, ON, Canada), and rabbit polyclonal anti-human melanopsin (OPN4; Abnova, Taipei, Taiwan) antibodies. Mouse monoclonal anti-chicken actin (Abcam Inc., Toronto, ON, Canada) was used as a loading standard. First, proteins were extracted from caudal fins of parrs and smolts (n = 5 per stage) by homogenization in ice-cold cell lysis buffer (50 mM Tris [pH 8.0], 150 mM NaCl, 1% Triton X-100; [Sigma–Aldrich, Oakville, Ontario, Canada]). Subsequently, lysates were centrifuged at 16,000 × g for 10 min at 4°C. Supernatants were collected and used in western blots with the antisera. Proteins were resolved in a 10% SDS-polyacrylamide gel and transferred to a PVDF membrane. After blocking using 5% non-fat dry milk in PBST (PBS with 0.1% Tween20) for 30 minutes at room temperature, membranes were incubated with antibodies (anti-UVS = 1:1000, anti-RH1 = 1:50,000, anti-Cry 1 = 1:500, anti-OPN4 = 1:20, and anti-actin = 1:5000, diluted in blocking solution) at 4°C overnight. Following several washes with PBST, immunosignals were detected with goat anti-rat, anti-mouse or anti-rabbit antibodies conjugated to alkaline phosphatase (Vector Laboratories, Burlingame, CA, USA) and visualized with BCIP/NBT (Calbiochem, San Diego, CA, USA).

## Supplementary Material

Supplementary Material
