# Supplementary material for: Ontogeny of melanophore photosensitivity in rainbow trout (Oncorhynchus mykiss)
Source: Biol Open. 2014 Oct 10;3(11):1032–6. doi: 10.1242/bio.201410058 (PMC4232760; doi:10.1242/bio.201410058)
Supplement: Supplementary Material [file supp_3_11_1032__index.html]

Ontogeny of melanophore photosensitivity in rainbow trout (Oncorhynchus mykiss) — Supplementary Material 

# Ontogeny of melanophore photosensitivity in rainbow trout (*Oncorhynchus mykiss*)

## bio.201410058 Supplementary Material

**Files in this Data Supplement:**

- Supplementary Material - Shyh-Chi Chen et al. doi: 10.1242/bio.201410058
