# Supplementary material for: Ontogeny of melanophore photosensitivity in rainbow trout (Oncorhynchus mykiss)
Source: Biol Open. 2014 Oct 10;3(11):1032–6. doi: 10.1242/bio.201410058 (PMC4232760; doi:10.1242/bio.201410058)
Supplement: Supplementary Material [file supp_bio.201410058_bio.201410058-s1.pdf]

Biology Open (2014) 000, 1–5 doi:10.1242/bio.201410058

**Supplementary Material**
**Shyh-Chi Chen et al. doi: 10.1242/bio.201410058**

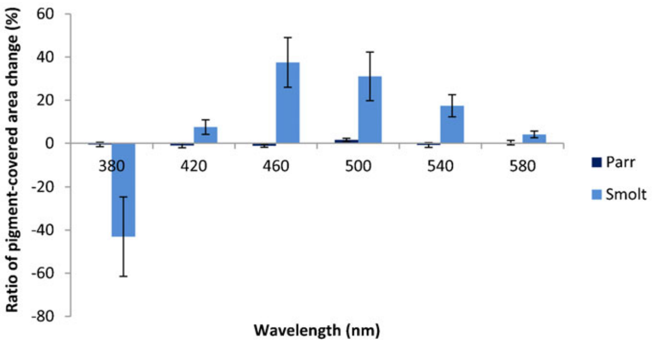


**Fig. S1. The photoresponses of parr and smolt melanophores.** Parr and smolt melanophores showed distinct responses to light. Smolt melanophores displayed aggregation and dispersion of melanosomes at different test wavelengths. By contrast, parr melanophores did not show obvious photoresponses.

**Table S1. Melanophore cell counts from the region (200 μm$^2$) between the 3$^{rd}$ and 4$^{th}$ caudal fin rays of parrs and smolts**

| Parr | | Smolt | |
|---|---|---|---|
| Fish ID | Cell counts | Fish ID | Cell counts |
| P-1 | 2 | S-1 | 22 |
| P-2 | 2 | S-2 | 19 |
| P-3 | 3 | S-3 | 15 |
| P-4 | 4 | S-4 | 20 |
| P-5 | 3 | S-5 | 8 |
| P-6 | 2 | S-6 | 13 |
| P-7 | 0 | S-7 | 9 |
| P-8 | 2 | S-8 | 15 |
| P-9 | 5 | S-9 | 18 |
| P-10 | 1 | S-10 | 24 |
| P-11 | 2 | S-11 | 18 |

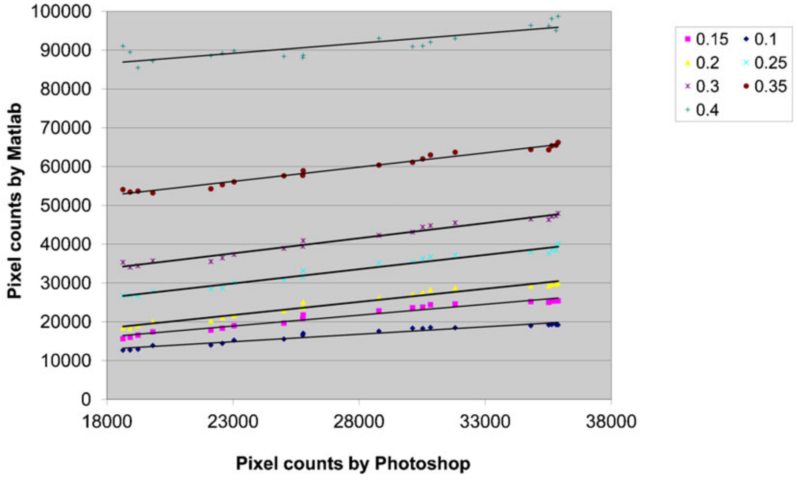


**Fig. S2. The correlation of pixel counts between Photoshop and Matlab measuring methods.** Melanophores (n=20) were manually selected with the selection tool in Photoshop software and the number of pixels within this region of interest was counted. For the measurement using Matlab, different thresholds were applied to the same cells and their pixel counts were compared with the result obtained using Photoshop. Threshold (TH) with the highest R squared value was chosen to be used in all measurements (R squared value: TH0.1=0.9488; TH0.15=0.9619; TH0.2=0.972; TH0.25=0.9706; TH0.3=0.9774; TH0.35=0.9793; TH0.4=0.7489).
